# Association between ICU admission (neutrophil + monocyte)/lymphocyte ratio and 30-day mortality in patients with sepsis: a retrospective cohort study

**DOI:** 10.1186/s12879-023-08680-4

**Published:** 2023-10-18

**Authors:** Manliang Guo, Wanmei He, Xueyan Mao, Yuling Luo, Mian Zeng

**Affiliations:** 1 Department of Medical Intensive Care Unit, The First Affiliated Hospital, Sun Yat-sen University, No.58 Zhongshan Road 2, Guangzhou, 510080 Guangdong China; 2 Institute of Pulmonary Diseases, Sun Yat-sen University, Guangzhou, Guangdong China

**Keywords:** Sepsis, Prognosis, NMLR, 30-day mortality, Inflammatory biomarker, MIMIC-IV

## Abstract

**Background:**

Sepsis is an important public health issue, and it is urgent to develop valuable indicators to predict the prognosis of sepsis. Our study aims to assess the predictive value of ICU admission (Neutrophil + Monocyte)/lymphocyte ratio (NMLR) on the 30-day mortality of sepsis patients.

**Methods:**

A retrospective analysis was conducted in septic patients, and the data were collected from Medical Information Mart for Intensive Care IV (MIMIC-IV). Univariate and multivariate Cox regression analyses were conducted to investigate the relation between ICU admission NMLR and 30-day mortality. Restricted cubic spline (RCS) was performed to determine the optimum cut-off value of ICU admission NMLR. Survival outcomes of the two groups with different ICU admission NMLR levels were estimated using the Kaplan-Meier method and compared by the log-rank test.

**Results:**

Finally, 7292 patients were recruited in the study, of which 1601 died within 30 days of discharge. The non-survival group had higher ICU admission NMLR values than patients in the survival group (12.24 [6.44–23.67] vs. 8.71 [4.81–16.26], P < 0.001). Univariate and multivariate Cox regression analysis demonstrated that ICU admission NMLR was an independent prognostic predictor on 30-day mortality (Univariate: P < 0.001; multivariate: P = 0.011). The RCS model demonstrated the upturn and non-linear relationship between ICU admission NMLR and 30-day mortality (Nonlinearity: P = 0.0124). According to the KM curve analysis,30-day survival was worse in the higher ICU admission NMLR group than that in the lower ICU admission NMLR group (Log rank test, P < 0.0001).

**Conclusion:**

The elevated ICU admission NMLR level is an independent risk factor for high 30-day mortality in patients with sepsis.

**Supplementary Information:**

The online version contains supplementary material available at 10.1186/s12879-023-08680-4.

## Background

Sepsis is a life-threatening condition characterized by physiologic, pathologic, and biochemical abnormalities induced by infection [1]. The incidence of sepsis is very high, and it remains an important public health issue and a huge burden across all economic regions [2]. Although many parameters are available for the diagnosis and evaluation of sepsis, it is also imperative to develop valuable indicators to predict the prognosis of sepsis. Patients with sepsis have an uncontrolled immune response and protracted inflammation due to overwhelming infection [3]. Inflammatory and immune cell counts are inexpensively measured and readily available parameters of inflammatory responses that can be obtained from whole blood count assays [4].

Many studies have indicated that blood cell ratios are valuable biomarkers with which to measure systemic inflammation and predict the prognosis of some health disorders, such as cardiovascular diseases [5, 6]. The ratio of the sum of the peripheral neutrophil and monocyte counts to the peripheral lymphocyte count (NMLR) is an indicator of the inflammatory and immune status. Some studies have suggested that the NMLR is a prognostic indicator for inflammatory and immune disorders [7, 8] and acute myocardial infarction [9]. However, evidence supporting the correlation between the NMLR at the time of admission to the intensive care unit (ICU) and the 30-day mortality of patients with sepsis remains scarce. Therefore, we conducted the present study to explore the relationship between the ICU admission NMLR and 30-day mortality of patients with sepsis.

## Methods

### Data source

The data used in our study were extracted from the Medical Information Mart for Intensive Care IV (MIMIC-IV version 1.0) database, which contains the hospitalization information of patients admitted to the Higher Medical Center in Boston, MA, USA from 2008 to 2019. We finished the required “Data or Specimens Only Research” course to use the database and obtained the corresponding certificate (Record ID: 11,347,834). Informed consent from individual patients was not required because the patients’ private information was recoded.

### Participants and data extraction

Data were extracted from the MIMIC-IV database using Navicat Premium 15, which was installed to run structure query language (SQL), and the code was obtained from the MIMIC Code Repository (https://github.com/MIT-LCP/mimic-code). Patients aged > 18 years who were admitted to the ICU and diagnosed with sepsis were included in this retrospective study. According to the Sepsis-3 criteria, sepsis was confirmed in patients with a Sequential Organ Failure Assessment (SOFA) score of ≥ 2 points accompanied by infection [10]. And septic shock was defined as sepsis with persisting hypotension requiring vasopressors to maintain MAP ≥ 65 mmHg and serum lactate level > 2 mmol/L (18 mg/dL) despite adequate volume resuscitation [1]. Patients were excluded from the study when repeated ICU stays were recorded and when NMLR data were missing. The following patient information was extracted: age, sex, race, hematocrit level, hemoglobin concentration, platelet count, white blood cell count, anion gap, bicarbonate, blood urea nitrogen, calcium, chloride, creatinine, glucose, sodium, potassium, monocyte count, neutrophil count, lymphocyte count, heart rate, systolic blood pressure, diastolic blood pressure, mean blood pressure, respiratory rate, temperature, peripheral oxygen saturation, renal replacement therapy on first day, SOFA score, systemic inflammatory response syndrome score, congestive heart failure, diabetes, hypertension, obstructive pulmonary disease, and peripheral vascular disease. The severity scores and all blood sample parameters were collected on the first day of ICU admission. The death time minus ICU discharge time was defined as survival time, the patient whose survival time less than 30 days was confirmed died within 30 days after ICU discharge.

### Statistical analysis

The data were analyzed using R version 4.2.1. The Kolmogorov–Smirnov test was used to assess the normality of the data distribution. Continuous variables are presented as median [interquartile range] and were compared using the Kruskal–Wallis test, whereas categorical variables are presented as count (percentage) and were compared using the chi-square test or Fisher’s exact test. We conducted univariate and multivariate Cox regression analyses to identify the risk factors for 30-day mortality of patients with sepsis. A restricted cubic spline was used to determine the cut-off value and visualize the nonlinear relationship between the ICU admission NMLR and 30-day mortality. Kaplan–Meier analysis was used to compare the survival status of two groups with different ICU admission NMLR levels.

## Results

### Participant characteristics

In total, 7292 participants were included in the study (4191 [57.5%] men; median age, 67.82 [56.58, 79.08] years). Of these 7292 patients, 1601 died within 30 days after discharge. The patients’ baseline characteristics across survival status strata are shown in Table 1. Patients in the non-survival group had higher ICU admission NMLR values than those in the survival group (12.24 [6.44, 23.67] vs. 8.71 [4.81, 16.26], respectively; P < 0.001). The most common comorbidity was hypertension (42.3%), followed by congestive heart failure (27.2%) and diabetes mellitus (22.6%). In addition, many other variables also exhibited statistically significant differences between survivors and non-survivors: age, ethnicity, hematocrit level, hemoglobin concentration, platelet count, anion gap, bicarbonate, BUN, calcium, chloride, creatinine, potassium, WBC, neutrophil, lymphocyte, heart rate, SBP, DBP, MBP, temperature, respiratory rate, SpO2, SOFA, SIRS, renal replacement therapy on first day, obstructive pulmonary disease.

**Table 1:** Baseline characteristic of the sepsis patients

Variables	Survivor(n = 5691)	Non-survivor(n = 1601)	*P*-value
Age (years)	66.47 [55.41, 77.48]	73.96 [61.16, 83.31]	**< 0.001**
Gender (male, n [%])	3286 (57.7)	905 (56.5)	0.402
Ethnicity (white, n [%])	3639 (63.9)	953 (59.5)	**< 0.001**
Hematocrit (%)	29.30 [25.10, 33.90]	28.80 [24.20, 34.00]	**0.021**
Hemoglobin (g/dL)	9.70 [8.20, 11.20]	9.30 [7.80, 10.90]	**< 0.001**
Platelet (K/µL)	166.00 [113.00, 235.00]	157.00 [93.00, 238.00]	**< 0.001**
Anion Gap (mEq/L)	13.00 [11.00, 15.00]	14.00 [12.00, 17.00]	**< 0.001**
Bicarbonate (mEq/L)	21.00 [19.00, 24.00]	19.00 [15.00, 23.00]	**< 0.001**
BUN (mg/dL)	19.00 [13.00, 32.00]	30.00 [18.00, 49.00]	**< 0.001**
Calcium (mg/dL)	8.00 [7.50, 8.50]	7.90 [7.30, 8.40]	**< 0.001**
Chloride (mEq/L)	102.00 [98.00, 106.00]	100.00 [96.00, 105.00]	**< 0.001**
Creatinine (mg/dL)	1.00 [0.70, 1.50]	1.30 [0.80, 2.10]	**< 0.001**
Glucose (mg/dL)	109.00 [93.00, 132.00]	109.00 [88.00, 140.00]	0.647
Sodium (mEq/L)	137.00 [134.00, 139.00]	136.00 [132.00, 140.00]	0.071
Potassium (mEq/L)	3.90 [3.50, 4.20]	3.90 [3.50, 4.40]	**< 0.001**
WBC (K/µL)	9.50 [6.50, 13.00]	10.70 [6.90, 15.20]	**< 0.001**
Monocyte (K/µL)	0.47 [0.27, 0.77]	0.50 [0.24, 0.83]	0.263
Neutrophil (K/µL)	8.82 [5.57, 12.94]	10.09 [6.08, 14.89]	**< 0.001**
Lymphocyte (K/µL)	1.02 [0.60, 1.61]	0.84 [0.46, 1.34]	**< 0.001**
NMLR	8.71 [4.81, 16.26]	12.24 [6.44, 23.67]	**< 0.001**
Heart rate (bpm)	85.12 [75.33, 96.61]	91.12 [78.33, 102.25]	**< 0.001**
SBP (mmHg)	113.28 [105.10, 124.42]	108.38 [100.19, 119.60]	**< 0.001**
DBP (mmHg)	60.75 [54.83, 67.73]	59.30 [52.76, 66.44]	**< 0.001**
MBP (mmHg)	75.30 [69.70, 82.04]	72.71 [66.71, 79.97]	**< 0.001**
Temperature (°C)	36.87 [36.63, 37.20]	36.74 [36.44, 37.11]	**< 0.001**
RR(insp/min)	19.00 [16.78, 21.84]	21.15 [18.18, 24.51]	**< 0.001**
SpO_2_ (%)	97.21 [95.84, 98.48]	96.72 [94.96, 98.28]	**< 0.001**
SOFA	3.00 [2.00, 4.00]	4.00 [2.00, 5.00]	**< 0.001**
SIRS	3.00 [2.00, 3.00]	3.00 [3.00, 4.00]	**< 0.001**
RRT(n[%])	333 (5.9)	172 (10.7)	**< 0.001**
CHF (n[%])	1545 (27.1)	438 (27.4)	0.893
Diabetes mellitus (n[%])	1296 (22.8)	350 (21.9)	0.461
Hypertension (n[%])	2428 (42.7)	655 (40.9)	0.221
Obstructive pulmonary disease(n[%])	593 (10.4)	116 (7.2)	**< 0.001**
Peripheral vascular disease(n[%])	573 (10.1)	147 (9.2)	0.316

**Notes:** P-values less than 0.05 is regarded statistically significant and shown in bold

**Abbreviations:** BUN, blood urea nitrogen; WBC, white blood cell; NMLR, (neutrophil + monocyte)/lymphocyte ratio; SBP, systolic blood pressure; DBP, diastolic blood pressure; MBP, mean blood pressure; SpO2, pulse oxygen saturation; SOFA, Sequential Organ Failure Assessment score; SIRS, Systemic Inflammatory Response Syndrome score; RR, respiratory rate; RRT, renal replacement therapy on first day; CHF, congestive heart failure

### ICU admission NMLR was an independent prognostic predictor of 30-day mortality in patients with sepsis

We conducted univariate and multivariate Cox regression analyses to investigate the relationship between the candidate risk factors and 30-day mortality. The hazard ratio (HR) and 95% confidence intervals (95% CIs) of the variables are shown in Table 2. The univariate Cox regression analysis showed that many variables were significantly associated with 30-day mortality of patients with sepsis. The variables associated with 30-day mortality (P < 0.05) were then subjected to the multivariate Cox regression analysis. The results showed that older age (HR,1.0236;95%CI,1.0198–1.0275;P < 0.001), higher anion gap (HR, 1.0541;95%CI,1.0399–1.0684;P < 0.001), higher BUN (HR,1.0082;95%CI,1.0059–1.0106;P < 0.001), higher ICU admission NMLR (HR,1.0036;95%CI,1.0008–1.0064;P = 0.011), higher heart rate (HR,1.0139;95%CI,1.01-1.0179;P < 0.001), higher respiratory rate (HR,1.0492;95%CI,1.0355–1.063;P < 0.001), higher SOFA (HR, 1.0934;95%CI,1.067–1.1206;P < 0.001) and higher SIRS (HR,1.143;95%CI,1.065–1.2268;P < 0.001) were independent risk factors for 30-day mortality. By contrast, higher hemoglobin concentration (HR,0.9634;95%CI,0.9395–0.9878;P = 0.004), higher platelet counts (HR,0.9992;95%CI,0.9987–0.9997;P = 0.003), higher creatinine (HR,0.8687;95%CI,0.8249–0.9149;P < 0.001), higher SBP (HR,0.9853;95% CI,0.9795–0.9912;P < 0.001), higher temperature (HR,0.7385;95%CI,0.6869–0.7940;P < 0.001), higher SpO2 (HR,0.9565;95%CI,0.9443–0.9689;P < 0.001) and obstructive pulmonary disease (HR,0.6033; 95%CI,0.4962–0.7335;P < 0.001) were protective factors. A higher NMLR was a risk factor in both the univariate analysis (HR, 1.0092; 95% CI, 1.0074–1.0111; P < 0.001) and multivariate analysis (HR, 1.0036; 95% CI, 1.0008–1.0064; P = 0.011). Next, the variables significantly associated with 30-day mortality in the multivariate Cox regression analysis were included in the proportional hazards assumption test, and variables considered as stratification factors were excluded. Finally, the following 13 variables constituted the multivariable-adjusted Cox regression model: platelet count, anion gap, NMLR, WBC, DBP, MBP, heart rate, SOFA, temperature, chloride, creatinine, potassium, obstructive pulmonary disease.

**Table 2:** Univariate and multivariate analysis of potential risk factors

Variables	Univariate analysis		Multivariate analysis	
	HR (95%CIs)	P-value	HR (95%CIs)	P-value
Age	1.0225(1.0191–1.0259)	**< 0.001**	1.0236(1.0198–1.0275)	**< 0.001**
Gender (male)	0.9548(0.8650–1.0540)	0.36		
Hematocrit	0.9943(0.9868–1.0019)	0.14		
Hemoglobin	0.9535(0.9320–0.9754)	**< 0.001**	0.9634(0.9395–0.9878)	**0.004**
Platelet	0.9992(0.9987–0.9997)	**< 0.001**	0.9992(0.9987–0.9997)	**0.003**
Anion Gap	1.1101(1.0994–1.1209)	**< 0.001**	1.0541(1.0399–1.0684)	**< 0.001**
BUN	1.0130(1.0115–1.0145)	**< 0.001**	1.0082(1.0059–1.0106)	**< 0.001**
Calcium	0.8880(0.8405–0.9382)	**< 0.001**	0.9961(0.9359–1.0602)	0.903
Chloride	0.9827(0.9761–0.9895)	**< 0.001**	0.9942(0.9870–1.0014)	0.115
Creatinine	1.0792(1.0538–1.1053)	**< 0.001**	0.8687(0.8249–0.9149)	**< 0.001**
Glucose	1.0016(1.0005–1.0027)	**0.0031**	1.0004(0.9993–1.0014)	0.505
Sodium	0.9953(0.9866–1.0041)	0.29		
Potassium	1.2338(1.1395–1.3359)	**< 0.001**	1.0766(0.9915–1.1690)	0.079
WBC	1.0093(1.0067–1.0119)	**< 0.001**	1.0020(0.9967–1.0073)	0.463
Monocyte	1.0156(0.9844–1.0479)	0.33		
Neutrophil	1.0266(1.0202–1.0330)	**< 0.001**	1.0054(0.9962–1.0147)	0.251
Lymphocyte	1 (0.9919–1.0082)	0.99		
NMLR	1.0092(1.0074–1.0111)	**< 0.001**	1.0036(1.0008–1.0064)	**0.011**
Heart rate	1.0166(1.0135–1.0196)	**< 0.001**	1.0139(1.01-1.0179)	**< 0.001**
SBP	0.9786(0.9750–0.9821)	**< 0.001**	0.9853(0.9795–0.9912)	**< 0.001**
DBP	0.9847(0.9799–0.9896)	**< 0.001**	0.9941(0.9810–1.0075)	0.386
MBP	0.9763(0.9712–0.9813)	**< 0.001**	1.0085(0.9923–1.0249)	0.308
Temperature	0.5916(0.5503–0.6360)	**< 0.001**	0.7385(0.6869–0.7940)	**< 0.001**
Respiratory rate	1.0973(1.0858–1.1089)	**< 0.001**	1.0492 (1.0355–1.063)	**< 0.001**
SpO_2_	0.9067(0.8980–0.9155)	**< 0.001**	0.9565(0.9443–0.9689)	**< 0.001**
SOFA	1.1618(1.1396–1.1844)	**< 0.001**	1.0934(1.067–1.1206)	**< 0.001**
SIRS	1.3702(1.2922–1.4529)	**< 0.001**	1.143(1.065–1.2268)	**< 0.001**
RR	1.8079(1.5433–2.1178)	**< 0.001**	1.1311(0.9268–1.3804)	0.226
CHF	1.0013(0.8971–1.1176)	0.98		
Diabetes mellitus	0.9495(0.8433–1.0689)	0.39		
Hypertension	0.9287(0.8406–1.0259)	0.15		
Obstructive pulmonary disease	0.7008(0.5801–0.8465)	**< 0.001**	0.6033(0.4962–0.7335)	**< 0.001**
Peripheral vascular disease	0.9127(0.7703–1.0815)	0.29		

**Notes:** P-values less than 0.05 is regarded statistically significant and shown in bold

**Abbreviations:** BUN, blood urea nitrogen; WBC, white blood cell; NMLR, (neutrophil + monocyte)/lymphocyte ratio; SBP, systolic blood pressure; DBP, diastolic blood pressure; MBP, mean blood pressure; SpO2, pulse oxygen saturation; SOFA, Sequential Organ Failure Assessment score; SIRS, Systemic Inflammatory Response Syndrome score; RR, respiratory rate; RRT, renal replacement therapy on first day; CHF, congestive heart failure

### Increased ICU admission NMLR was associated with higher 30-day mortality

**Fig. 1:**
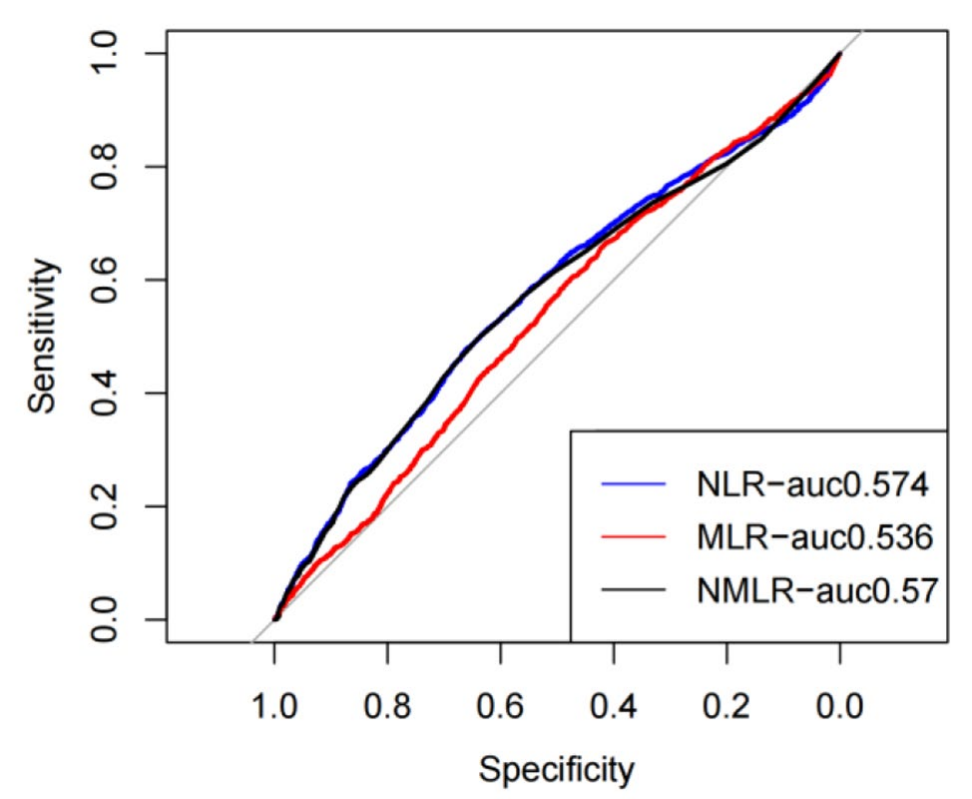
Receiver operating characteristic curve to evaluate the predictive value of NLMR, NLR and MLR for 30-day mortality of septic patients in ICU

We performed receiver operating characteristic curve (ROC) to evaluated the predictive value of NLMR for 30-day mortality in septic patients admitted to ICU and compared it to the performance of NLR and MLR (Fig. 1). The AUC value of the NLMR model was (0.574, 95% CI: 0.558–0.591), which was significantly higher than the AUC value of the MLR model (0.536, 95% CI: 0.520–0.553; p < 0.001) and the NLR model (0.563, 95% CI: 0.558–0.568; p < 0.05). Therefore, admission NMLR had better predictive value for 30-day mortality of septic patients.

**Fig. 2:**
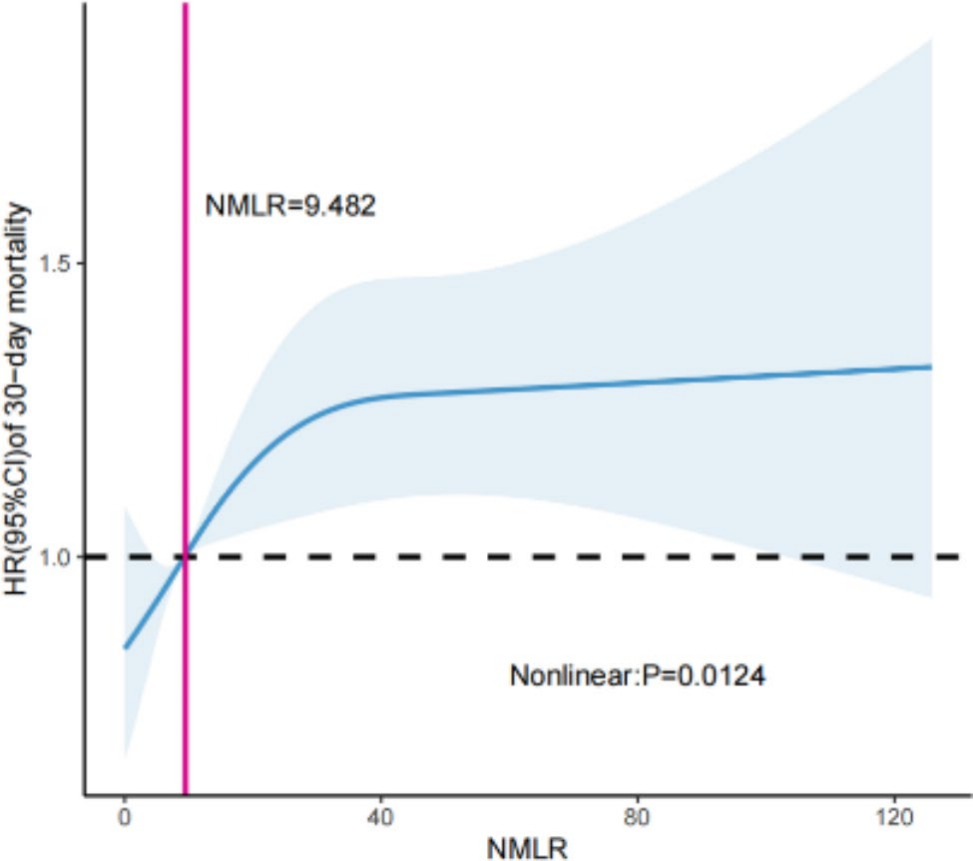
Restricted cubic spline curve to model the nonlinear relationship between the ICU admission NMLR and 30-day mortality

We used restricted cubic splines with four knots at the 5th, 35th, 65th, and 95th centiles to model the nonlinear relationship between the ICU admission NMLR and 30-day mortality. The model was adjusted for the above 13 cofounders that accorded with the proportional hazard assumption. As shown in Fig. 2, the ICU admission NMLR was nonlinearly associated with 30-day mortality of patients with sepsis (*P* = 0.0124); the HR increased rapidly when the ICU admission NMLR was > 9.482 and then reached a plateau when the ICU admission NMLR was approximately 40. In general, the HR of 30-day mortality increased as the ICU admission NMLR increased.

**Fig. 3:**
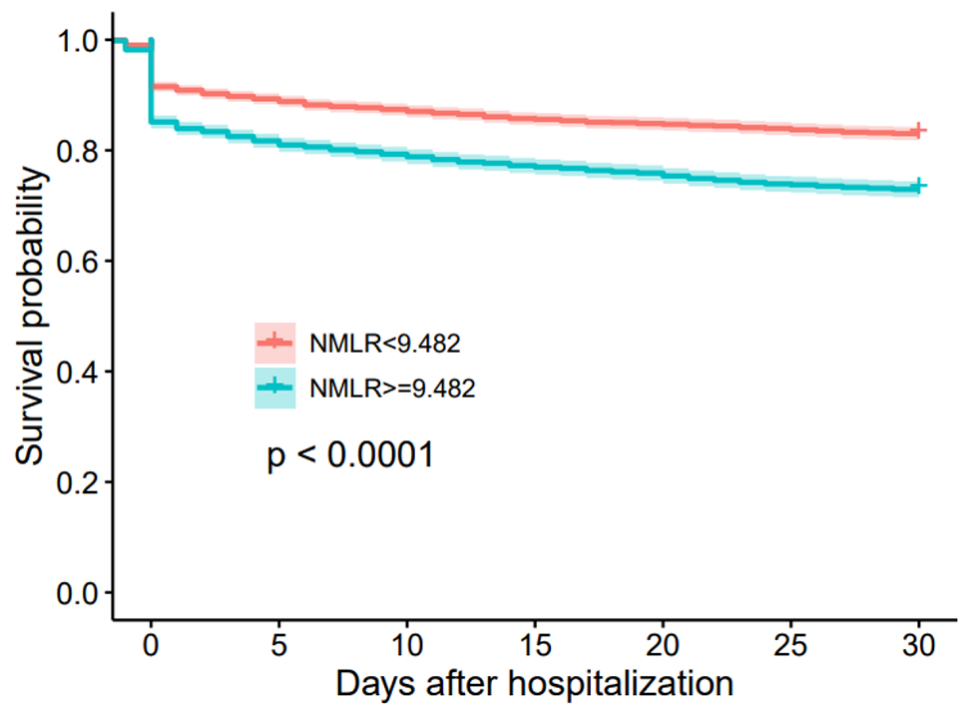
Kaplan-Meier plots for 30-day mortality by ICU admission NMLR strata

Next, we divided the study population into a higher ICU admission NMLR group (NMLR > 9.482) and a lower ICU admission NMLR group (NMLR < 9.482) according to the cut-off point. We then performed a Kaplan–Meier analysis between the two groups. As shown in Fig. 3, the survival curve in the higher ICU admission NMLR group was significantly lower than that in the lower ICU admission NMLR group (log-rank test, P < 0.0001). Thus, a higher ICU admission NMLR was associated with increased 30-day mortality.

## Discussion

In the current study, we discovered that elevated ICU admission NMLR level was associated with increased risk of mortality in patients with sepsis. We observed that ICU admission NMLR was nonlinearly associated with 30-day mortality of septic patients and had better predictive value for prognosis of septic patients. In detail, NMLR was a protective indicator when below 9.482 (HR < 1), but it turned to be a risk indicator when NMLR > 9.482 (HR > 1). Moreover, Kaplan–Meier analysis revealed that higher ICU admission NMLR was associated with increased 30-day mortality of septic patients.

Uncontrolled immune response and the development of inflammatory disorders are very common in patients with sepsis. Monocytes, neutrophils, and other innate immune cells release proinflammatory cytokines that result in dysregulated inflammatory responses, causing systemic damage [11]. In addition to this innate hyperinflammatory response, patients with sepsis also endure persistent immunosuppression, which may be responsible for the high mortality rate after being discharged. Lymphopenia occurs after sepsis [12], monocytes and macrophages become vulnerable to mounting a proinflammatory response [13, 14], and neutrophil dysfunction increases the release and activation of immunosuppressive myeloid-derived suppressor cells [12]. These cells coordinately mediate immunosuppression by releasing anti-inflammatory cytokines such as interleukin 10 in patients with sepsis [14]. Therefore, indicators that comprehensively reflected the immune cell activation status had important predictive value for the prognosis of septic patients.

The NMLR is an indicator of the inflammatory and immune status, and it demonstrates the balance of neutrophils/monocytes and lymphocytes in the systemic inflammatory response [15]. Thus, the NMLR has been widely studied with respect to its prognostic value for diseases such as hepatocellular carcinoma, cardiovascular diseases, and multiple myeloma. Liao et al. [15] revealed that the NMLR could better predict the postoperative recurrence-free survival and overall survival of patients with hepatocellular carcinoma than other predictive factors. Yan et al. [8] found that adding the NMLR to the Canada Acute Coronary Syndrome Risk Score model significantly improved the model efficiency, and the NMLR could effectively predict the cardiovascular mortality in very old patients with acute myocardial infarction. Pang et al. [7] reported that a lower NMLR (< 1.90) was an independent prognostic factor for progression-free survival as well as early immune reconstruction and a lower disease burden in patients with multiple myeloma who were treated with bortezomib + cyclophosphamide + dexamethasone regimen therapy. In addition, NMLR also had better predictive value for in-hospital mortality in patients with acute myocardial infarction than other indicators such as NLR, neutrophils, monocytes and lymphocytes [9]. Our study also demonstrated that NMLR had advantages in predicting the prognosis of septic patients compared to NLR and MLR. The current study was the first to investigate the relationship between the ICU admission NMLR and sepsis-related mortality. The results showed that patients with sepsis in the non-survival group had a higher ICU admission NMLR value than those in the survival group (12.24 [6.44, 23.67] vs. 8.71 [4.81, 16.26], respectively; P < 0.001). In addition, the ICU admission NMLR was independently associated with 30-day mortality in the univariate and multivariate Cox regression analyses. Moreover, the restricted cubic spline model and Kaplan–Meier analysis revealed that a higher ICU admission NMLR was associated with higher 30-day mortality of patients with sepsis.

The present study had some limitations. First, the laboratory data used in the study were collected on the first day of ICU admission; thus, we could not analyze the continuous change in the NMLR. Second, selection bias and confounding bias were unavoidable in this retrospective observational study. Third, higher NMLR might also had directive value for the management of sepsis, such as predicting septic shock [12.38(9.70,15.06) vs18.25(16.83,19.67), P < 0.001] and the need for vasopressor therapy [12.13(9.53,14.73) vs. 16.22(13.58,18.86), P < 0.001], which deserves further investigation. Finally, the data in our study were extracted from a single-center MIMIC IV database and therefore may not be representative enough. Future larger multicenter prospective cohorts are needed to prove or disprove this study findings. More future analysis taking into consideration the changing course of the patients throughout their stay in the hospital and the change in the NMLR accordingly.

## Conclusion

As a result, the present study demonstrated that NMLR was a simple and valuable biomarker related to mortality of patients with sepsis. Patients with ICU admission NMLR not less than 9.482 should be treated more carefully as they are more likely to have an adverse prognosis.

### Electronic supplementary material

Below is the link to the electronic supplementary material.

Supplementary Material 1

## Data Availability

The datasets generated and/or analyzed during the current study are available in the physionet repository(https://physionet.org/content/mimiciv/1.0/).

## Abbreviations

BUN: blood urea nitrogen
WBC: white blood cell
NMLR: (neutrophil + monocyte)/lymphocyte ratio
SBP: systolic blood pressure
DBP: diastolic blood pressure
MBP: mean blood pressure
SpO2: pulse oxygen saturation
SOFA: Sequential Organ Failure Assessment score
SIRS: Systemic Inflammatory Response Syndrome score
RR: respiratory rate
RRT: renal replacement therapy on first day
CHF: congestive heart failure
